# Balancing act: how Apelin tunes vascular and haemogenic identities

**DOI:** 10.1038/s44319-026-00696-6

**Published:** 2026-01-26

**Authors:** Rui Monteiro

**Affiliations:** 1 Department of Cancer and Genomic Sciences, University of Birmingham, Birmingham, UK; 2 Birmingham Centre for Genome Biology, University of Birmingham, Birmingham, UK

**Keywords:** Signal Transduction, Stem Cells & Regenerative Medicine, Vascular Biology & Angiogenesis

## Abstract

New research in EMBO Reports identifies a function for Apelin signaling in balancing angiogenesis, vascular maintenance and haematopoiesis.

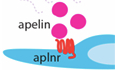

## Teaser

Haematopoietic stem and progenitor cells (HSPCs) maintain haematopoiesis throughout life. Their formation occurs early in embryonic development and is regulated by many intrinsic and extrinsic factors that delicately balance the need to maintain a vascular network with the need to generate HSPCs de novo. Most extrinsic factors such as BMP and Notch act instructively in haemogenic endothelial cells to induce HSPC fates. A new study by Eberlein et al (2025) identifies a key role for Apelin signalling acting indirectly by limiting the number of arterial endothelial cells that become haemogenic, independently of Notch, BMP or Wnt signalling (Eberlein et al, 2025). Arterial endothelial cells that do not respond to Apelin more frequently convert to the haemogenic endothelial cell fate, giving rise to higher numbers of haematopoietic stem and progenitor cells in the embryo that persist into adulthood. This work highlights a critical temporal window where Apelin functions as a rheostat, balancing angiogenesis, vascular maintenance and haematopoiesis.

**Figure 1:**
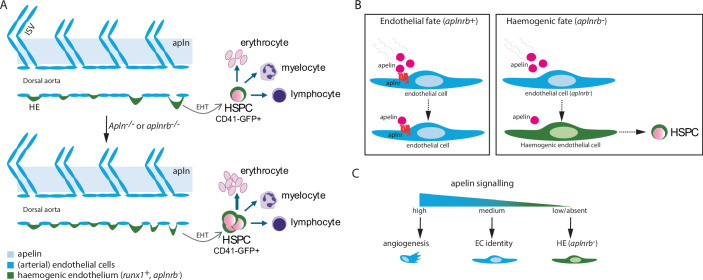
Overview of Apelin activity in endothelial cells in embryonic angiogenesis and haematopoiesis. (**A**) Schematic representation of the increase in haemogenic endothelial cells (HECs, green cells) upon ablation of *aplnrb* or *apln* in zebrafish. (**B**) The presence of *aplnrb* maintains endothelial cell (EC) identity, whereas its absence from HEC enables the transition to HSPCs. (**C**) A rheostat model correlating the level of Apelin activity to EC or HEC cell fate. ISV intersegmental vessels, EHT endothelial-to-haematopoietic transition.

Endothelial cell (EC) differentiation is a fundamental process that is required for both blood vessel formation and the establishment of the haematopoietic system. HSPCs arise from a specialised subset of arterial cells located in the floor of the embryonic dorsal aorta, the haemogenic endothelium (Bertrand et al, 2010; Kissa and Herbomel, 2010). Apelin signalling in endothelial cells is mediated by the G-coupled Apelin receptor b (*aplnrb*) and transduced by MEK/ERK and PI3K/AKT pathways to regulate endothelial migration, sprouting, and vascular patterning (Cox et al, 2006). While the role of Apelin signalling in angiogenesis is well established, its role in haematopoiesis is much less clear. Earlier work suggested that loss of Apelin signalling leads to a decrease in HSPC output (Jackson et al, 2021) from differentiated mouse embryonic stem cells in vitro, and addition of exogenous APLN to mouse aorta-gonad-mesonephros (AGM) explants also led to impaired generation of HSPCs. Here, Eberlein and colleagues (Eberlein et al, 2025) identify a new role for Apelin signalling in regulating whether endothelial cells follow an angiogenic or haemogenic route in the ventral wall of the dorsal aorta, the birthplace of HSPCs (Bertrand et al, 2010; Kissa and Herbomel, 2010). This novel role for Apelin signalling is mediated by its *aplnrb* receptor, which they demonstrate is present in ECs but absent from haemogenic endothelial cells (HECs) fated to become HSPCs. Using confocal imaging and clever genetics, the authors go on to show that loss of *apln* or of its receptor, *aplnrb*, leads to increased HSPC output (Fig. 1A). *Aplnrb*^−^ ECs become HECs and go on to form HSPCs, whereas *aplnrb* + ECs retain endothelial identity (Fig. 1B). Thus, EC fate is governed by the presence or absence of the Aplnrb receptor; in its absence, dorsal aorta ECs undergo fate conversion from (arterial) ECs to HECs. Interestingly, previous work from the Zilong Wen lab suggests that practically all of the dorsal aorta floor cells are HECs (Zhao et al, 2022), suggesting that the role of Apelin is more nuanced. Indeed, the EC to HEC fate conversion by loss of Apelin signalling is limited, reflected by a ~1.5-fold increase in HSPC numbers in embryos, and a similar increase in progenitor numbers in adult animals. Note that while more HECs were produced, the authors do not report the presence of ectopic HECs in the posterior cardinal vein, indicating that the competence to respond to haemogenic-inducing signals is independent of Apelin signalling.

Taken together with previous work from the authors and others in the field, the data strongly support that Apelin signalling could be viewed as a rheostat rather than a binary switch. High Apelin activity promotes angiogenesis, intermediate activity maintains arterial EC identity, and its absence permits hemogenic specification and HSPC emergence (Fig. 1C). This continuum is evident from zonation of *aplnrb* expression and the graded phenotypes in loss- and gain-of-function experiments. While some discrepancies remain between different studies on the role of Apelin in haematopoiesis, the study by Eberlein and colleagues underscores a common theme: the indirect nature of Apelin signalling in haematopoiesis, acting in the endothelial microenvironment (Jackson et al, 2021) rather than in HSPCs themselves.

## Erythroid shift in *aplnrb*^−/−^ HSPCs

Analysis of the haematopoietic output indicated a shift to an erythroid fate in embryos and adult maternal-zygotic *apln* (*Mzapln)* mutants, suggesting that the levels of Apelin signalling perceived by haemogenic endothelial cells can modulate their differentiation capacity. This is consistent with HSPC heterogeneity previously documented by others (Ghersi et al, 2023; Xia et al, 2021) and suggests an early role for Apelin signalling in contributing to this heterogeneity. Further analysis of gene expression programmes in early HE/arterial ECs in *aplnrb* or *apelin* mutants (by e.g., single-cell RNAseq) will be informative to identify the molecular changes underlying the shift in lineage potential. Intriguingly, while in the embryo only the erythroid lineage is over-represented, adult *MZapln* mutants also display increased numbers of myeloid lineage cells (neutrophils, macrophages), suggesting a potentially distinct role for Apelin signalling after HE/HSPC cell fate has been initially established. A more detailed understanding of the potential later role for Apelin signalling might help reconcile the data from Eberlein et al with the observed increase in myeloid differentiation in murine AGM explants upon activation of the APLN pathway (Jackson et al, 2021).

## Upstream regulators of *aplnrb* expression

An unresolved question is which factors determine how *aplnrb* expression is turned off to enable HE commitment to HSPC fate? Eberlein et al tested known extrinsic regulators of HSPC but found that neither Wnt, Notch, Shh, nor BMP signalling (Sugden and North, 2021) played a role in regulating *aplnrb*expression, indicating that other potential regulators may be involved. Candidates include other pathways with identified roles in HSPC formation, such as adenosine signalling and TGFβ signalling (Sugden and North, 2021). Testing these pathways, combined with careful analysis of *aplnrb* regulatory regions, might identify regulators of *aplnrb* expression in HECs and potentially enable modulation of haematopoietic output in vitro for therapeutic purposes. Given that *aplnrb* is expressed in all ECs earlier in development (Helker et al, 2020), regulation likely involves an inhibitory factor that is upregulated during HE commitment, or silencing of an activator of *aplnrb* expression as cells transition to a haemogenic fate.

In summary, Eberlein et al uncover a previously unappreciated role for Apelin signalling as a quantitative regulator of endothelial fate, acting to fine-tune HSPC output. This work provides a conceptual framework for how vascular maintenance and haematopoiesis are balanced during development. The discovery that Apelin signalling indirectly limits hemogenic conversion, coupled with its influence on lineage bias, opens new avenues for future research—both to dissect the molecular mechanisms controlling *aplnrb* downregulation and to explore whether modulating Apelin activity could be leveraged to expand or tailor HSPC production for regenerative therapies.
